# Taking on the Commercial Determinants of Health at the level of actors, practices and systems

**DOI:** 10.3389/fpubh.2022.981039

**Published:** 2023-01-04

**Authors:** Jennifer Lacy-Nichols, Alexandra Jones, Kent Buse

**Affiliations:** ^1^Melbourne School of Population and Global Health, The University of Melbourne, Parkville, VIC, Australia; ^2^The George Institute for Global Health, Sydney, NSW, Australia; ^3^The George Institute for Global Health, Imperial College, London, United Kingdom

**Keywords:** power, public health, corporation, action, heuristic, systems

## Abstract

Tackling the Commercial Determinants of Health (CDoH) is necessary for progress on health equity and will determine whether or not the health-related targets of the SDGs are met. We present a simple visual heuristic of three core aspects of CDoH: commercial actors, commercial practices, and system-level dynamics (which commercial actors influence and perpetuate). We use this heuristic to highlight key research gaps, in particular the need for more voices and evidence on CDoH from the Global South, particularly on what works to curb harmful impacts. We also propose an agenda to address CDoH and actions for different stakeholders. While efforts to curb specific commercial practices are important, far more attention and effort are needed at the systems level, as they can fundamentally shift the way power is distributed in society to improve health equity.

## Introduction

Evidence has been accumulating for decades on how the Commercial Determinants of Health (CDoH) undermine health equity, and it is increasingly clear that they threaten progress across the health-related Sustainable Development Goals (SDGs). In 2021, several new frameworks and definitions on CDoH were published, the World Health Organization (WHO) announced a new program of work to address CDoH, multilateral organizations, including the United Nations (UN) and the International Monetary Fund (IMF), urged action on commercial entities, and a global tax on transnational corporations (TNCs) was negotiated (1–3). COVID-19 has opened a political window, and many governments have taken enormously progressive steps forward to support health equity. The time to accelerate further action on CDoH is now.

As the CDoH field seeks to consolidate knowledge and enable action, we offer a simple visual heuristic that segments CDoH into three levels: (1) commercial actors; (2) practices; and (3) systems. We use this heuristic to highlight key research gaps and to propose an agenda to address the CDoH.

## Understanding CDoH at three levels

**Figure 1:**
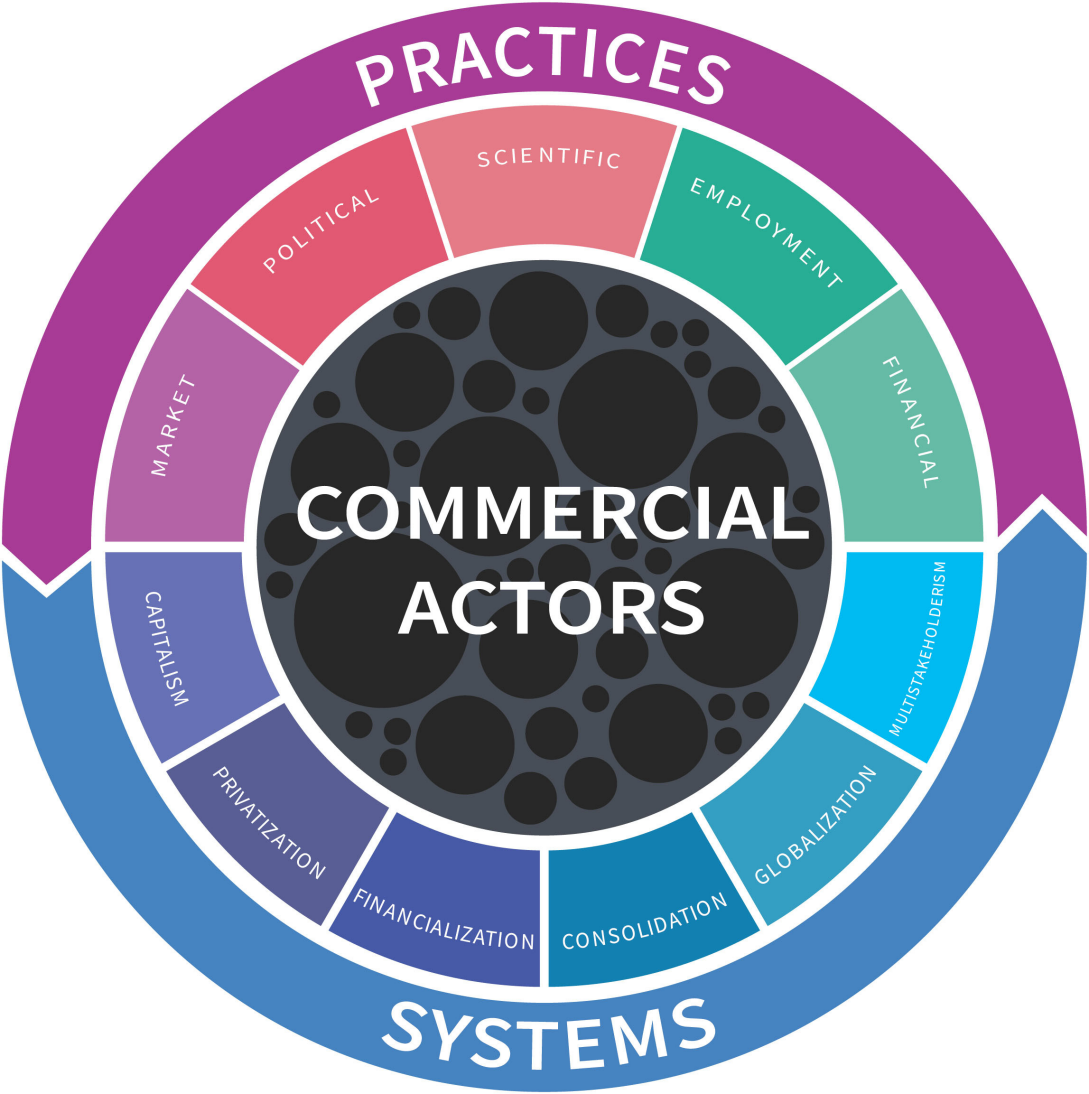
A visual heuristic of the CDoH.

While there are a myriad of definitions of the CDoH (4), most can be distilled to a core concept: the pathways through which commercial actors influence health. These pathways can be differentiated into downstream, meso-level commercial practices that influence communities and individuals, and upstream, macro-level systems in which commercial entities act (1). These two categories are inherently connected: powerful commercial actors actively shape systems in their interests, and systems enable practices that further entrench and increase that power. Figure 1 illustrates these three key dimensions of CDoH.

Commercial actors are at the center of the CDoH concept and are represented by circles of varying sizes indicating their diversity. The commercial sector is heterogeneous in its makeup and health impacts. Commercial actors differ according to their resources, for example. market share, revenue, employees, geographic and planetary footprint, political connections, industry associations, etc. These resources readily translate into power to shape market and political systems in their favor and the capacity to take advantage of pro-capitalism system-level dynamics. While some of the most powerful businesses are transnational corporations (TNCs), there are also trusts, sole enterprises, cooperatives and partnerships. State-owned enterprises blur the line between public and private, and not-for-profits and charitable foundations with commercial funding blur the line between private sector and civil society. The commercial sector produces a huge number of goods and services, including those potentially harmful to health (e.g., firearms, pharmaceuticals, automobiles, gambling, social media) as well as privatized goods and services such as education, healthcare, utilities, transport (5, 6). CDoH research thus far has focused on a small segment of the commercial world selected for the harmful products they produce, such as tobacco, alcohol and ultra-processed foods (often referred to as “unhealthy commodity industries”). A focus on commercial actor resources could widen the gaze to include other TNCs from different sectors whose health impacts have been subject to less scrutiny.

Commercial practices are the more visible pathways through which commercial actors influence health, and include: marketing, political, science, employment and financial practices (see Box 1 for illustrative examples). The exploitative practices of TNCs are especially worthy of scrutiny, as they can deepen and entrench existing inequities in income, health and life expectancy, including disparities between the Global North and South (7). This further stymies efforts for Low- and Middle-Income Countries (LMICs) to develop their own economies and escape the legacy of neo-colonialism on the health of their populations.

Box 1 **Illustrative commercial actor practices which influence health (see Appendix 1 for sources).**

**Market:** Poorly regulated “buy now pay later” companies (e.g., AfterPay, Zip, Affirm, and others) have been criticized for predatory marketing linked to rising consumer debt—the industry includes retail, healthcare and housing.

**Political:** Google has more than 258 instances of “revolving door” activity in the United States, including White House officials, the Department of Justice and the Federal Trade Commission—the same agencies tasked with investigating antitrust.

**Scientific:** Coca-Cola and the International Life Sciences Institute have funded research to support the soft drink industry's message that physical activity, not diets, is the key driver of obesity.

**Employment:** The commercialization of the incarceration system, often referred to as the Prison Industrial Complex, has led to the exploitation of often minority populations for dangerous and virtually unpaid labor, while migrant workers and their children are subject to violence, abuse, hazardous living conditions and have limited access to healthcare and education.

**Financial:** The “Big Four” accounting firms—PwC, Deloitte, KPMG and EY—play a significant role in defending and enabling systematic tax avoidance, which depletes public resources that might otherwise be used to promote public health.

Unfettered capitalism underpins and enables negative CDoH (8). This system-level dynamic plays out through a myriad of forces. The consolidation and globalization of commercial actors has concentrated resources and power amongst a small number of companies. Of the world's 100 largest economies, around 70 are now corporations not countries, and the top 50 companies constitute around 30% of global GDP (9, 10). Foreign direct investment and trade agreements have expanded the presence of TNCs around the world, a process linked with poorer health outcomes and rising inequities (11). Financialization repositions social services as lucrative investment opportunities, where goals focus on short-term profits rather than equity. The financialization of healthcare, for example, has exposed essential medicines to market volatility, leading to wildly fluctuating costs that are unaffordable to those most in need. Business partnerships within the UN system have proliferated, bringing with them opportunities to influence the ideological, political, technical agendas, as well as value-orientation, of those organizations and their programs (12, 13). While proponents of “multistakeholderism” argue that it brings a diversity of opinions and resources to the table through more participatory governance arrangements, poorly governed multistakeholderism reifies existing power differentials—both between commercial actors and civil society, and also between commercial actors and less well-resourced states (14). These dynamics collectively enable powerful commercial actors to promote business interests over health and equity.

## Approaches to tackle CDoH and protect public health: From corporate practices to systemic transformation

Our heuristic points to three broad approaches to addressing the CDoH that begin with a narrow focus on specific commercial actors and/or their commodities, and progressively broaden to encompass a wider range of practices and ultimately systems (see Box 2 for illustrative examples). Action at the system level is fundamentally more upstream, and for this reason has the greatest impact whilst also being the most politically challenging (1).

Box 2 **Approaches to tackle CDoH at the levels of actors, practices and systems (see Appendix 1 for sources).**

**Actors**

- Implement taxes on tobacco, alcohol, and sugary drinks or other health-harming products.
- Restrict predatory marketing and implement front-of-pack warning labels on tobacco, alcohol and ultra-processed foods.

**Practices**

- Mandate employee benefits (including paid parental leave, unemployment benefits and sick leave), including for casual and contract workers.
- Regulate profit repatriation, where a parent company avoids paying taxes in its own jurisdiction by first shifting profits to one of its subsidies in a low-tax jurisdiction and then “lending” its parent company back the same money.

**Systems**

- Develop and enforce strict transparency and disclosure requirements for public servants and politicians at all levels of government about engagement with commercial actors, e.g., real-time disclosure of political donations, gifts, hospitality and meetings with government officials.
- Implement progressive corporate taxation, wealth or “solidarity” taxes to more equitably redistribute wealth.
- Earmark corporate taxes to support public goods, e.g., research, independent media, etc.
- Mandate greater human rights accountability on TNCs for exploitative labor practices and environmental degradation.
- Develop and enforce rigorous conflict of interest (COI) standards for engagement with commercial and quasi commercial actors, including regulation governing the revolving door.
- Use antitrust suits to break up monopoly industries (for example, the technology industry in US).
- Embed a health lens in investment decisions—Tobacco Free Portfolios is one example, which could be expanded to incorporate other health and equity metrics.
- Establish an intergovernmental tax commission to negotiate a global minimum tax floor to address the “race to the bottom” where countries sacrifice environmental standards and human rights in pursuit of lucrative investments.
- Implement and enforce existing access and benefit sharing mechanisms (such as the Nagoya Protocol and the Pandemic Influenza Preparedness Framework) to ensure that intellectual property is not protected at the expense of national sovereignty, equitable access to vaccines, or other “benefits.”
- (Re)municipalize public goods and services such as water, energy, the postal system, etc.

Most efforts to tackle the CDoH focus on specific commodities (especially tobacco, alcohol and foods). Many of these are considered “NCD Best Buys” interventions by WHO and include restrictions on marketing and promotion, access to policy making, participation in scientific research along with regulations requiring product warning labels, taxes, age and place restrictions, and health campaigns (15). While there is a considerable evidence base demonstrating efficacy, these initiatives alone are inadequate to tackle the system-level dynamics that enable commercial harms.

Initiatives at the level of commercial practices tend to be implemented at local or national levels of government (and it is crucial that these initiatives are government-led, not voluntary, industry-led initiatives). Thus, it is important to recognize that countries will have differential capacity and power to both adopt and effectively implement and enforce such regulations due to existing structural limitations (e.g., a reliance on revenue from foreign direct investment) (16). See Box 2 for examples.

System-level changes are about rebalancing the power disparities amongst powerful economic actors and other stakeholders. Beyond efforts to restrict and redistribute resource concentration, it is also important to support alternative forms of business organization such as cooperatives, which may have more democratic or inclusive forms of decision-making. Some countries are exploring “wellbeing economy” principles, which challenge the ideological dominance of gross domestic product as the primary marker of progress and instead prioritize social justice and a healthy planet (17). Rebalancing power also involves ensuring spaces for civil society and other non-commercial parties to participate in governance (12, 13). To support these efforts will require challenging the ideological dominance of capitalism, neoliberalism, multistakeholderism and other pro-commercial values, and the narratives that sustain them, while reaffirming the importance of democratic accountability.

## Opportunities to reshape systems in support of health equity

Multiple actions by multiple stakeholders are necessary to create an enabling environment to curb the negative CDoH. We highlight five priority actions in Box 3 and expand on these below.

Box 3 **Priority actions to advance a CDoH agenda.**

**Governments**: Develop a five-year strategy for action on CDoH with list of priorities for a multisector program of work.

Multilaterals: Rebalance participation in governance fora to ensure actors from civil society organizations and the Global South have a voice.

**Civil society**: Build coalitions and foster public support for ambitious and effective government regulation of CDoH.

**Researchers**: Expand our understanding of the system-level dynamics enabling CDoH—including opportunities to shift these to foster health promoting forms of commerce and share this evidence widely outside academic publications.

**WHO**: Develop technical guidance on COIs for governments, multilaterals, NGOs, and others that encompasses commercial and quasi-commercial actors.

### Governments

There are two immediate tasks for governments. First, enact policies and regulations to curtail the market and political influence of commercial actors (see Box 2 for examples). This can include limits on and transparency around commercial participation in politics, anti-trust legislation and progressive taxation on corporations. New and existing South-South alliances can share relevant knowledge and resources and bolster the capacity of LMICs to confront the CDoH. The Open Government Partnership, which helps civil society and governments collaborate on developing action plans with accountability mechanisms embedded, provides a further approach (18). Second, provide sustainable and independent funding for research on CDoH, including an independently managed public repository of information on CDoH and specific commercial actors (19). Health levies on harmful commodities (such as tobacco) can provide one source of funding—investments from sovereign wealth funds or pension funds are another possibility.

### Multilaterals

The multilateral system can play three important roles. First, it can provide national and global leadership in the CDoH field. WHO can lead by example by establishing rigorous standards for its own engagement with commercial and quasi-commercial actors, including for staff secondments from commercial and quasi-commercial actors (such as consulting firms). This could include a process for voluntary contributions to go into a blind pool, thereby limiting the ability of donors to dictate how funds are used and provide the WHO with greater autonomy. These actions are relevant for other UN agencies and multilaterals more broadly who address the social, and commercial, determinants of health (13). Second, it must ramp up development of standards and technical guidance. This could include guidance on governance mechanisms (such as co- or public regulation) and principles to safeguard against conflicts of interest and promote accountability (such as ambitious targets, transparent reporting, independent monitoring and remedial action on progress) (20). WHO's program of work on the Economic and Commercial Determinants of Health offers a platform to disseminate information and generate political attention to these issues (3). Third, it should better support countries to adopt and implement rigorous standards to hold commercial actors to account. This could include capacity building activities, such as those undertaken by the Pan-American Health Organization to support the implementation of the WHO tool to prevent and manage COI in nutrition programs in the Americas (21).

### Civil society

Civil society will be critical to systems transformation and has two central tasks. First, it can provide a watchdog function. Civil society already monitors and reports on commercial actors, and NGOs such as Open Secrets, Transparency International, Corporate Europe Observatory, and InfluenceMap publicize, share and interpret information made public by governments or leaked. Second, it can lead and grow advocacy coalitions to foster demand for change. Campaigns that shine a light on commercial practices can target company reputations and lead to substantial changes, such as companies ending their relationships with controversial trade associations like the American Legislative Exchange Council. Fracturing these coalitions is an important strategy to weaken the structural power of commercial actors and limit their opportunity to influence decision-making (22).

### Researchers

We envision four contributions that the research community can make. First, we challenge researchers to look beyond “unhealthy commodity industries” to other sectors (especially privatized public goods and services). CDoH research must also develop a richer understanding of different business models, including TNCs, but also alternatives models such as cooperatives or social enterprises. Second, there is much work to do in exploring CDoH in LMICs, as many TNCs base the production components of their supply chains in these countries yet base their headquarters and key leadership in high-income countries (often where the majority of taxes are paid). An important challenge moving forward will be to build up the resources of research institutions in LMICs so that they can lead research in this space. Third, effective interventions will require more nuanced frameworks and typologies to differentiate between commercial actors and consider quasi-commercial organizations (such as philanthropic foundations) or privatized services (such as healthcare). An independently managed public repository would support these efforts and enable researchers, governments and policy makers to access evidence on CDoH (19). Finally, we call on researchers to identify approaches to limit the harms perpetuated by commercial actors, including the narratives which normalize the systems described above (23) and how these can be implemented in practice.

### Independent media

Independent media can contribute in two ways. First, to draw attention to the CDoH and disseminate research findings widely. And secondly, to foster public outrage over commercial malfeasance and increase public support for government action. One proposal to fund independent news is a tax on large technology companies which have cannibalized much of the advertising revenue. The International Federation of Journalists has proposed that governments should implement a digital services tax on the largest technology companies to create a global fund for independent journalism (24).

### Commercial and quasi-commercial actors

The commercial sector itself has a potentially important role to play. For example, the financial sector can incorporate a health lens in their investment and divestment decision-making to reallocate funding away from actors engaging in harmful practices to support more equitable forms of commerce. A range of financing initiatives support small and medium enterprises (SMEs) in LMICs, often with an explicit focus on supporting women entrepreneurs to access capital, such as the Women Entrepreneurship Development Project in Ethiopia and the Access to Finance for Women SMEs Project in Bangladesh (25). While incremental in nature, these actions present the opportunity for small wins and a vision of economies not characterized by unfettered capitalism.

## Conclusion

To comprehensively address the CDoH requires nothing short of a fundamental restructure of the global political and socio-economic system. Progress, at times, feels bleak. We hope that our heuristic can aid in making the CDoH more accessible and help identify entry points to this complex, fundamental and urgent public health challenge. The Lancet Series on Commercial Determinants of Health, from which some of the ideas and the heuristic in this article are developed, provides more detailed information, models and frameworks to guide the development of responses to the harmful impacts of commercial entities on health and health equity. These provide an important resource for future research, policy and advocacy and in protecting the health and wellbeing of communities from unfettered capitalism (26–28).

## Data availability statement

The original contributions presented in the study are included in the article/Supplementary material, further inquiries can be directed to the corresponding author.

## Author contributions

JL-N, AJ, and KB conceived the article and jointly developed the outline. JL-N initially drafted the paper, with all co-authors substantially contributing to its revision and finalization. All authors contributed to the article and approved the submitted version.
