# Corrigendum: Taking on the Commercial Determinants of Health at the level of actors, practices and systems

**DOI:** 10.3389/fpubh.2023.1305512

**Published:** 2023-11-16

**Authors:** Jennifer Lacy-Nichols, Alexandra Jones, Kent Buse

**Affiliations:** ^1^Melbourne School of Population and Global Health, The University of Melbourne, Parkville, VIC, Australia; ^2^The George Institute for Global Health, Sydney, NSW, Australia; ^3^The George Institute for Global Health, Imperial College, London, United Kingdom

**Keywords:** power, public health, corporation, action, heuristic, systems

In the published article, there was an error in the **Conclusion** section, *Protecting public health from unfettered capitalism*, in which concluding statements and references were erroneously omitted.

This section previously stated:

“To comprehensively address the CDoH requires nothing short of a fundamental restructure of the global political and socio-economic system. Progress, at times, feels bleak. We hope that our heuristic can aid in making the CDoH more accessible and help identify entry points to this complex, fundamental and urgent public health challenge.”

The corrected section appears below:

“To comprehensively address the CDoH requires nothing short of a fundamental restructure of the global political and socio-economic system. Progress, at times, feels bleak. We hope that our heuristic can aid in making the CDoH more accessible and help identify entry points to this complex, fundamental and urgent public health challenge. The Lancet Series on Commercial Determinants of Health, from which some of the ideas and the heuristic in this article are developed, provides more detailed information, models and frameworks to guide the development of responses to the harmful impacts of commercial entities on health and health equity. These provide an important resource for future research, policy and advocacy and in protecting the health and wellbeing of communities from unfettered capitalism (1–3).”

A correction has also been made to include **Acknowledgments**, which appear below, as this section was erroneously omitted in the originally published article:

“This perspective builds on the Lancet Series on Commercial Determinants of Health (CDoH). The visual heuristic comprising commercial actors, practices and systems is inspired by the model (in which the authors of this paper played no role) and framework developed in paper 1 and 2, respectively, of the Lancet Series as well as by discussions of the co-authors leading the Series. We would like to particularly acknowledge the work of the series' lead authors Anna Gilmore and Sharon Friel and series' chair Rob Moodie.”

This perspective was based partly on ideas generated during the development and writing of the Lancet Series on Commercial Determinants of Health (CDoH). It drew, in particular, on the model and ideas in Paper 1 in that Series. JL-N had full access to all papers in that series. AJ was involved only in paper 2 of that series. KB was not involved in the series or aware of its contents. In the original perspective, JL-N did not acknowledge the influence of the Lancet Series on CDoH, and the authors have corrected this with additional text, references and an acknowledgement section. JL-N apologizes for this error.

The authors apologize for these errors and state that they do not change the scientific conclusions of the article in any way. The original article has been updated.
