# Supplementary material for: Taking on the Commercial Determinants of Health at the level of actors, practices and systems
Source: Front Public Health. 2023 Jan 4;10:981039. doi: 10.3389/fpubh.2022.981039 (PMC9872788; doi:10.3389/fpubh.2022.981039)
Supplement: Supplementary file 1 [file Data_Sheet_1.docx]

**Appendix 1**

**Box 1 sources**

Nguyen T. 2021. Buy now, pay later changed retail. Health care and rent are next. Vox [Internet]. 26 November 2021. Available from: <https://www.vox.com/the-goods/2021/5/11/22429014/buy-now-pay-later-pandemic-expansion>

Campaign for Accountability. 2021. Google's Revolving Door (US). *Tech Transparency Project*. [https://www.techtransparencyproject.org/articles/googles-revolving-door-us. Accessed 22 July 2021](https://www.techtransparencyproject.org/articles/googles-revolving-door-us.%20Accessed%2022%20July%202021)

Greenhalgh, S. 2019. Making China safe for Coke: how Coca-Cola shaped obesity science and policy in China. 364

Klein D, Lima J. 2021. The Prison Industrial Complex as a Commercial Determinant of Health. American Journal of Public Health.111(10):1750-2

UNICEF. 2018. Report reveals major challenges facing children in construction site camps.15 December 2021. Available from: <https://www.unicef.org/thailand/press-releases/report-reveals-major-challenges-facing-children-construction-site-camps>

Elbra A, Mikler J, Murphy-Gregory H. 2020. Knowledge and power: the role of the Big Four in the competitive disharmonization of global corporate tax avoidance regulations. In: Mikler J, Ronit K, editors. MNCs in Global Politics: Edward Elgar Publishing.

**Box 2 sources**

ShareAction. Investors waking up to the need for ESG overhaul on health2021 12 November 2021. Available from: <https://shareaction.org/news/investors-waking-up-to-the-need-for-esg-overhaul-on-health>

Institute on Taxation and Economic Policy. Comprehensive Guide to “Repatriation” Proposals 2016 29 November 2021. Available from: <https://itep.org/comprehensive-guide-to-repatriation-proposals/>

OECD. Lobbying in the 21st Century: Transparency, Integrity and Access 2021 2 December 2021. Available from: <https://doi.org/10.1787/c6d8eff8-en>

World Health Organization. Tackling NCDs:'best buys' and other recommended interventions for the prevention and control of noncommunicable diseases2017 21 December 2021. Available from: <https://apps.who.int/iris/handle/10665/259232>

PAHO. Preventing and Managing Conflicts of Interest in Country-level Nutrition Programs: A Roadmap for Implementing the World Health Organization’s Draft Approach in the Americas2021. Available from: <https://iris.paho.org/handle/10665.2/55055>

Ryding T. As G20 meets to rubberstamp OECD tax deal, 130+ developing countries push for UN tax body. Eurodad [Internet]. 2021 16 November 2021. Available from: <https://www.eurodad.org/g77_global_tax_body_un>

Transnational Institute. Reclaiming Public Services: How cities and citizens are turning back privatisation2017 12 February 2022. Available from: https://www.tni.org/files/publication-downloads/reclaiming_public_services.pdf;
